# Controllable Preparation of Fused Silica Micro Lens Array through Femtosecond Laser Penetration-Induced Modification Assisted Wet Etching

**DOI:** 10.3390/ma17174231

**Published:** 2024-08-27

**Authors:** Kaijie Cheng, Ji Wang, Guolong Wang, Kun Yang, Wenwu Zhang

**Affiliations:** 1 Center of Materials Science and Optoelectronics Engineering, University of Chinese Academy of Sciences, Beijing 100049, China; chengkaijie@nimte.ac.cn (K.C.);; 2 Research Center for Laser Extreme Manufacturing, Ningbo Institute of Materials Technology & Engineering, Chinese Academy of Sciences, Ningbo 315201, China; wangguolong@nimte.ac.cn; 3 College of Mechanical Engineering, Zhejiang University of Technology, Hangzhou 310014, China

**Keywords:** micro lens array, femtosecond-laser-induced modification, wet etching, imaging, fused silica

## Abstract

As an integrable micro-optical device, micro lens arrays (MLAs) have significant applications in modern optical imaging, new energy technology, and advanced displays. In order to reduce the impact of laser modification on wet etching, we propose a technique of femtosecond laser penetration-induced modification-assisted wet etching (FLIPM-WE), which avoids the influence of previous modification layers on subsequent laser pulses and effectively improves the controllability of lens array preparation. We conducted a detailed study on the effects of the laser single pulse energy, pulse number, and hydrofluoric acid etching duration on the morphology of micro lenses and obtained the optimal process parameters. Ultimately, two types of fused silica micro lens arrays with different focal lengths but the same numerical aperture (NA = 0.458) were fabricated using the FLPIM-WE technology. Both arrays exhibited excellent geometric consistency and surface quality (Ra~30 nm). Moreover, they achieved clear imaging at various magnifications with an adjustment range of 1.3×~3.0×. This provides potential technical support for special micro-optical systems.

## 1. Introduction

With the continuous development of modern optoelectronic technology, the demand for device integration, miniaturization, and multifunctionality in professional micro optical systems (MOSs) is becoming increasingly urgent. Among them, the micro lens arrays (MLAs), as a key optical component, can provide benefits, such as compact and lightweight designs, multifunctionality, and exceptional integration capability [1,2,3], for MOS systems, thus receiving increasing attention. The MLAs find applications across various domains, including optical imaging [4,5,6], emerging energy technologies [7,8], and cutting-edge display systems [9,10].

In the field of advanced technological applications, the fabrication techniques for MLAs have faced increasingly stringent demands. Traditional methods for MLA fabrication include photolithography [11,12,13], thermal reflow [14], electron beam lithography [15], and self-assembly [16]. However, photolithography and electron beam lithography are costly, complex in the process, and inefficient in production. Thermal reflow exhibits poor flexibility, low processing precision, and complex procedures, and is primarily applicable to polymers [17,18]. Fused silica is a typical hard and brittle material, which has excellent chemical stability, mechanical properties, and an extremely low coefficient of thermal expansion compared with materials like polydimethylsiloxane (PDMS) and polymethyl methacrylate (PMMA). Moreover, it boasts high transparency in the ultraviolet-to-near-infrared spectrum and an exceptionally stable refractive index, making it an ideal material for MLAs used in MOSs. Nevertheless, its high stability and fragility pose challenges for achieving high-precision processing.

With the development of laser technology, ultrafast lasers have emerged as an effective method for micro/nano fabrication [19,20,21,22]. Femtosecond (Fs) lasers have the advantages of extremely short pulses (~10^−15^ s), high peak power (10^22^ W/cm^2^), and strong controllability, which allows them to direct writing and modify materials within transparent materials [23]. However, it is difficult to avoid thermal effects during the direct etching of quartz using a femtosecond laser, which will affect the quality and surface characteristics of the processing, hindering the creation of high-quality MLAs. For a long time, how to further improve the surface quality and preparation controllability of MLAs has been a challenge faced by researchers. Therefore, this limits the application of femtosecond laser processing as a single technological means in the manufacturing of micro lens arrays.

In recent years, researchers have improved many new processes based on femtosecond lasers. Liu et al. [19] successfully prepared uniformly arranged MLAs on a silicon surface using femtosecond laser-assisted plasma-etching technology (FLPE). However, this method exhibits extremely high sensitivity to experimental conditions and is costly, which poses great challenges for industrial applications. Femtosecond-laser-induced modification-assisted wet etching (FLIM-WE) has high processing efficiency, a low cost, and relatively relaxed environmental requirements [24,25,26], making it a more ideal method. This method is based on the deposition of femtosecond laser energy inside the material, which leads to a phase transition of the material. Due to the extremely high peak power of femtosecond lasers, atoms or molecules in materials absorb light energy and undergo nonlinear optical effects, such as photoionization and thermalization [27,28]. These effects can cause local high temperatures and pressures in materials, resulting in phenomena, such as densification, defect formation, and changes in the absorption rate [29,30]. The modified area can achieve rapid and high-quality structural etching through corrosion of the corresponding solvent. Currently, the FLIM-WE method has been widely used by researchers for the preparation of MLAs with a high surface quality [31,32,33]. Feng Chen et al. [34] fabricated a sub-millimeter-sized MLA on a silicon dioxide substrate with a variable NA, using femtosecond laser linear scanning modification-assisted wet etching. Generally speaking, the size of micro lenses is closely related to the area of the laser-modified region. Nevertheless, the majority of existing research employs a top-down femtosecond laser processing approach. In the process, the preceding laser pulse induces modifications to the material’s surface, creating a previously modified layer. Defects, such as voids within this modified layer, can affect the absorption and scattering of subsequent laser pulses on the material’s surface, thereby impacting the controllability of the interaction between subsequent pulses and the material significantly. Therefore, challenges persist in achieving size controllability, and there is a lack of foundational process explorations.

In this paper, we proposed a method based on femtosecond laser back-facing penetrating single-point exposure for material modification. Specifically, we adopted a bottom-up approach to achieve back-facing penetration-induced modification. Subsequent pulses directly interact with the material without passing through the modified layer, which is induced by the initial pulse. This approach significantly enhanced the controllability of MLA fabrication. By analyzing the influence of laser parameters on inducing modified pores, we have identified the optimal process parameters. By changing the area of the modified region, the geometric morphology of the micro lens was altered to achieve the goal of controlling the NA. Then, combined with hydrofluoric acid (HF) etching, we have realized the controllable fabrication of fused silica MLAs with a fixed NA (0.458) and variable focal length efficiently, by optimizing the laser parameters and etching time. And both types of the MLAs with a size of around 40 microns demonstrated excellent imaging capabilities, highlighting the potential applications of this method in the preparation of special MOSs.

## 2. Experiment

### 2.1. Method and Material

**Figure 1:**
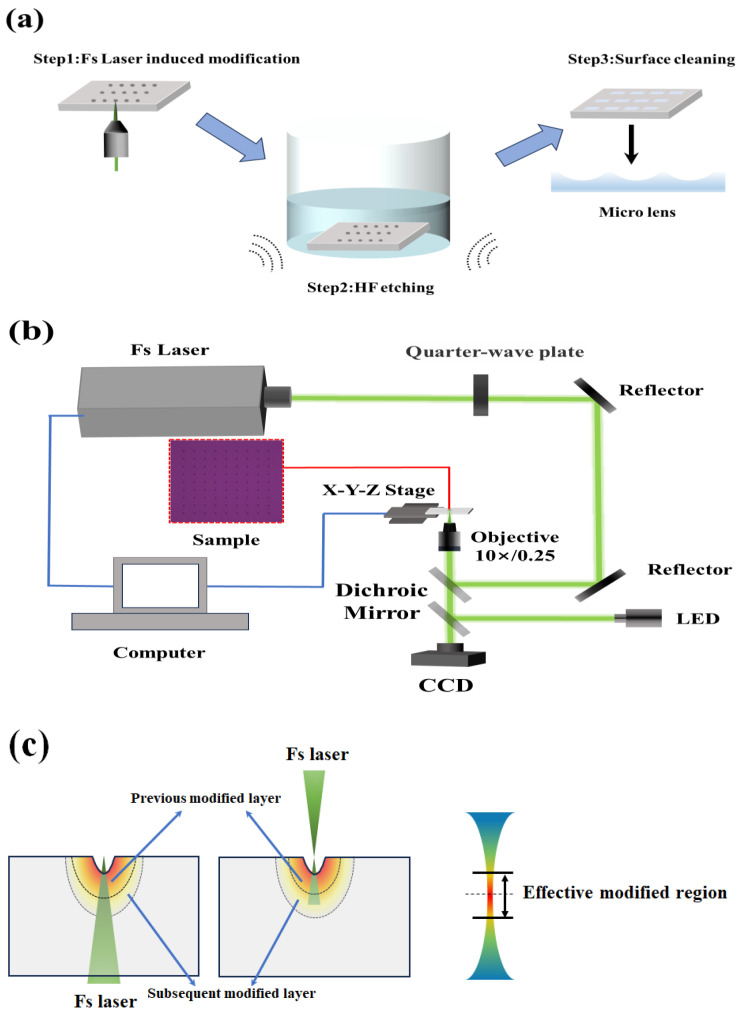
Experimental setup and method. (**a**) Steps in the fabrication of micro lens array. Step 1: using femtosecond laser-induced modification, step 2: place the modified sample in HF acid solution for ultrasonic-assisted wet etching, step 3: surface cleaning of MLA, the illustration pointed to by the black arrow shows a vertical-sectional schematic of the morphology of the microlens array; (**b**) schematic diagram of device femtosecond laser-induced modification of fused silica; (**c**) comparison between penetration FLPIM method and traditional top-down modification method.

The FLIM-WE preparation method for a micro lens array (MLA) mainly includes the following three steps, as shown in Figure 1a: femtosecond laser penetration-induced modification (FLPIM) of fused silica (step 1), wet etching assisted by a ultrasonic water bath to remove the modified surface layer (step 2), and subsequent surface cleaning (step 3).

Initially, the back facing of the fused silica sample underwent induced modification using femtosecond laser single-point exposure, which generated micro lens structures with each exposure. Then, HF acid solution was used to wet etch the modified micro lens structure. The concentration of etching solution used in the experiment was 20%. We mixed 15 mL of a hydrogen fluoride (40% concentration, analytical grade) solution and 15 mL of deionized water with a ratio of 1:1 in a polytetrafluoroethylene container. The modified samples were then immersed in the etching solution within a polytetrafluoroethylene container and subjected to wet etching under ultrasonic water bath conditions to form the final micro lens. In the experiment, the power of the ultrasonic equipment (JM-03D-80, Skymen, China) was set at 80 W, the ultrasonic frequency was 40 kHz, and the water bath temperature was 20 °C. Finally, the processed samples were subjected to ultrasonic cleaning to remove residual etching residues. During the whole experiment, the ambient temperature was controlled within 25 ± 2 °C, and the humidity was controlled within 50 ± 5%. In addition, the material used to prepare MLAs in this experiment was an ultraviolet fused silica glass thin plate (0.3 mm thickness, JGS1 brand).

### 2.2. Experimental Setup

The experimental setup for FLPIM is depicted in Figure 1b. The laser beam from the femtosecond laser (FemtoYL-Green, YSL Photonics, Wuhan, China, laser wavelength of 515 nm, pulse frequency of 25 kHz, pulse width of ~373 fs) passed through a quarter-wave plate, then through a reflection mirror group and a dichroic mirror, and entered into the microscope objective (10×, NA 0.25). The polarization state of the laser beam emitted by the laser was vertical polarization. We rotated the angle of the optical axis of the quarter wave plate to modulate linearly polarized light into circularly polarized light. The light beam focused by the microscope objective traveled from bottom to top, penetrating the interior of the sample. Then, the focus shifted to the back-facing surface of the sample. A CCD sensor was used for monitoring the laser exposure process and finding the focal plane. The sample moved downwards along the Z-axis. If the CCD observed two focal points, then the plane where the second focal point located is the back-facing upper surface to be induced for modification. This penetration-induced modification method will effectively avoid the absorption and scattering effects of the modified layer generated by the previous pulse on the subsequent pulse.

As shown in Figure 1c, we designed a bottom-up penetration-induced modification processing method. Firstly, the back surface of the sample is modified by a focused femtosecond laser to form induced modification holes. The material under the hole undergoes phase transition and defects, forming the previous modified layer. Next, the subsequent laser pulses continue to penetrate the material, forming a new layer of a modified area within it. However, in the traditional top-down surface treatment method, the following laser pulses inevitably need to pass through the previous modified layer and modified hole. Only then can the subsequent modified layer be formed inside the material. Obviously, this improved method effectively avoids the nonlinear effects of the previously modified layer on the subsequent pulses (including light absorption and scattering), thereby improving the controllability of the modification effect.

This device controlled the laser power used for inducing modification by adjusting the current through a computer. At the same time, the computer coordinated the movement of the X-Y-Z stage to adjust the pulse state of the emitted laser through the phase-sensitive optical (PSO) function. When the X-Y-Z stage completed the specified movement distance, PSO controlled the femtosecond laser to output pulses for the FLPIM process based on the pre-set current and pulse quantity by the computer.

Characterization of the fabricated micro lens morphology was performed using a laser scanning confocal microscope (LSCM, VK-X200, Keyence, Japan). The HF solution etching process lasted for 150 min, with samples retrieved for morphology observation and analysis every 60 min.

## 3. Results and Discussion

We designed experiments to investigate the effects of the laser pulse energy, pulse quantity, and HF acid etching duration on the geometric dimensions of the micro lens morphology, in order to find suitable process intervals. Then, we prepared MLAs based on process parameters and measured their size and micro imaging performance.

### 3.1. Influence of Laser Parameters on Induced Modified Holes

**Figure 2:**
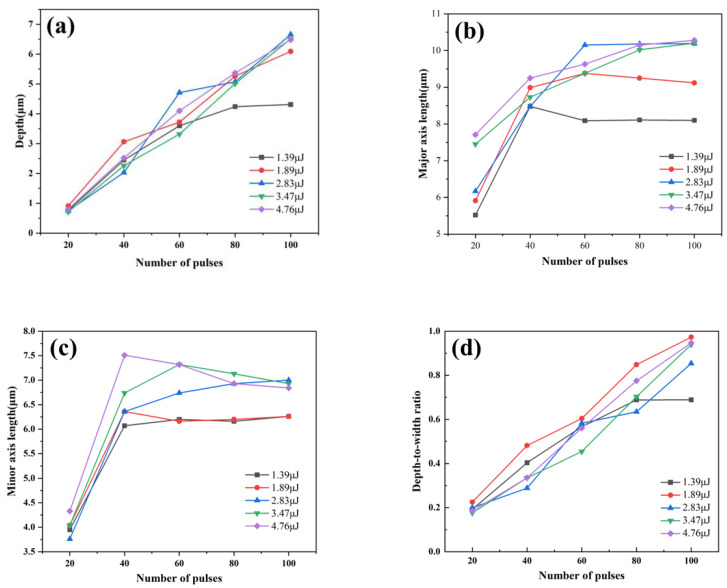
Influence of laser parameters on micro modified hole dimensions. (**a**) Variation in the depth of the modified holes; (**b**) variation in major axis length of the modified holes; (**c**) variation in minor axis length of the modified holes; (**d**) variation in depth-to-diameter ratio of the modified holes.

We investigated the impact of varying the number of laser pulses (20–100 pulses) and the energy during the manufacturing process of micro modified holes on fused silica surfaces, and presented our findings in Figure 2. The micro modified holes formed are elliptical in shape, with the length of the minor and major axes represented on the vertical axes of Figure 2b and Figure 2c, respectively.

Figure 2a illustrates that under identical energy conditions, the etching depth of the micro lens increases notably with higher pulse counts. Furthermore, with elevated pulse energies, the rate of etching depth augmentation also escalates proportionally. At a single pulse energy of 1.39 μJ, minimal changes in the etching depth are observed. Whereas at 1.89 μJ, the depth increases sharply from 0.91 μm to 6.09 μm with an increasing pulse count. This means that in order to achieve precise control of the modification depth, energy exceeding 1.89 uJ cannot be used. Nevertheless, the maximum etching depth does not escalate indefinitely with higher pulse numbers. Even with a pulse energy increase to approximately 2.5 times (from 1.39 μJ up to 4.76 μJ) at pulse numbers of 100, the maximum etching depth only marginally increased from 6.0 μm to 6.8 μm.

Figure 2b,c depict that when the pulse count is below 40, both the major and minor axis lengths of the micro lens significantly increase with additional pulses. However, 40 pulses are the turning point. When beyond 60 pulses, these dimensions stabilize. This phenomenon can be attributed to the widening of the micro-hole surface area, where the laser edge energy falls below the material ablation threshold, thereby reducing the fused silica-removal capability. Consequently, further dimensional changes cease despite continued laser exposure on the fused silica pits. That is to say, there is a non-linear relationship between the depth of modified pores and the number of pulses. As the number of pulses increases, the lateral dimension of the modified holes will gradually reach its limit.

Figure 2d reveals that at a single pulse energy of 1.39 μJ, when the number of pulses is less than 80, the depth-to-diameter ratio of the micro modified holes increases linearly with the pulse count. Meanwhile, micro holes modified with 80 and 100 pulses exhibit nearly identical depth-to-diameter ratios, approximately 0.69. At higher single pulse energies of 1.89 μJ and above, the depth-to-diameter ratio also increases linearly with the pulse count, approaching 0.9 at 100 pulses. However, a larger depth-to-diameter ratio for the micro lens implies reduced curvature, leading to diminished magnification in microscopic imaging and increased distortion of the original image. Addressing these challenges may necessitate additional optical design strategies to mitigate substantial distortion effects [35].

In conclusion, taking into account these observation results, our study has discovered excellent laser parameters, namely the use of lower pulse energy (1.39 μJ–1.89 μJ) and a moderate number of pulses (60–80) to prepare MLAs.

### 3.2. Influence of Wet Etching Time on the Formation of Micro Lenses

**Figure 3:**
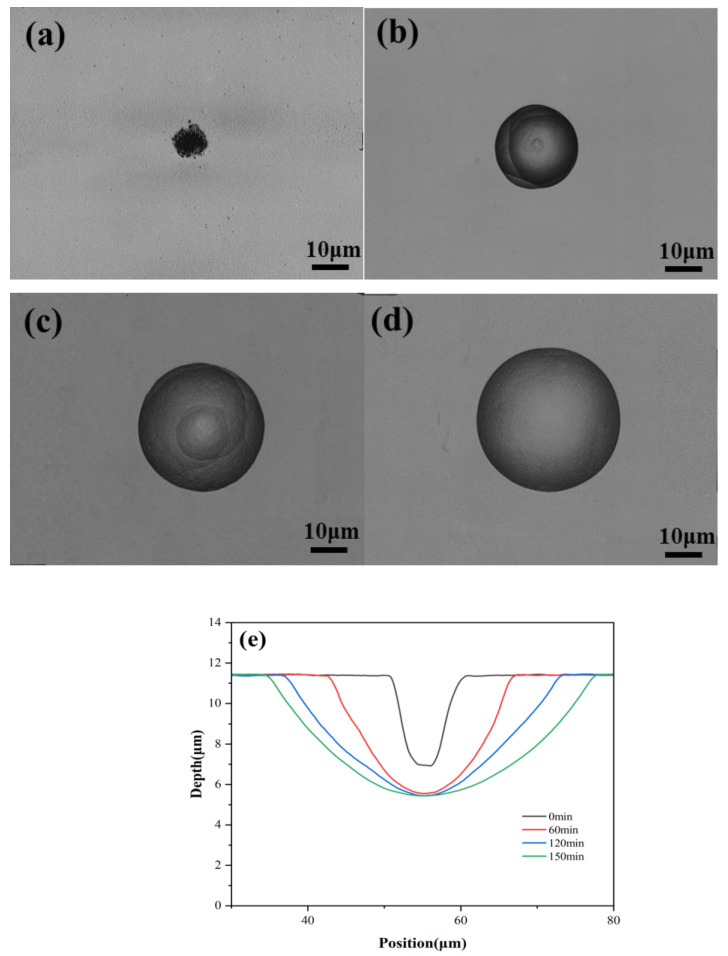
Morphological changes of micro lens under different HF solution etching times. (**a**) 0 min; (**b**) 60 min; (**c**) 120 min; (**d**) 150 min, scale bar in Figure 3a,b is 10 μm; (**e**) variation in micro lens contour profiles with the etching time.

According to Step 2, we explored the influence of the HF solution etching time on the dimensions and surface quality of the micro lens. Using a femtosecond laser with a single pulse energy of 1.39 μJ and 60 pulses, the material underwent modification, followed by observing the morphology changes of the micro lens after etching in the HF solution for different durations, as depicted in Figure 3. The etching times in Figure 3a–d are 0, 60, 120, and 150 min, respectively.

Initially, the morphology around the micro lens appears disorderly with a rough surface (Figure 3a). As the etching time progresses, the diameter of the micro lens gradually increases, approaching a perfect circular contour. Early in the etching process, rough striped structures are visible inside the micro lens (shown in Figure 3b,c). With prolonged etching durations, the etching process reaches saturation, and the internal striped structures gradually disappear, leading to a smoother surface morphology. This indicates that complete etching of the laser-modified region has occurred in the HF acid solution, when the etching time reaches 150 min.

Figure 3e illustrates the transition of the HF solution from an initially anisotropic to an isotropic behavior. Notably, significant differences in etching rates are observed between the regions modified via femtosecond laser treatment and those that remain unmodified. Regarding the contour width, the HF solution exhibits an efficient etching capability and rapid etching rate on the sidewalls formed through femtosecond laser modification. After 120 min, the modified areas begin to diminish, resulting in a gradual slowdown in the etching rate for the contour width. In terms of the etching depth, the modified region at the bottom of the micro lens is rapidly removed by the HF solution, achieving complete etching within the initial 60 min. As time went on, the etching depth hardly changed. Obviously, the etching rate in the vertical direction is much higher than that in the radial direction.

This difference is attributed to the fact that the femtosecond laser modification intensity is mainly concentrated in the vertical direction. the strongly induced modified region is primarily confined within the beam waist, resulting from the direct interaction between the femtosecond laser and the material. In contrast, the modification in the horizontal direction is mainly influenced by refracted light beams, which are scattered by the modified region in the vertical direction. Due to the attenuation of the energy of these refracted beams, the modification effect in the horizontal direction is not as strong as that in the vertical direction. Consequently, during etching with HF acid, a faster etching rate is observed in the vertical direction. As the etching progresses, the area exposed to the HF solution exhibits isotropic etching characteristics similar to the original material. At this point, wet etching has been completed. Only by achieving complete wet etching can micro lenses with extremely low surface roughness be obtained.

### 3.3. Controlled Fabrication of the Micro Lens

**Figure 4:**
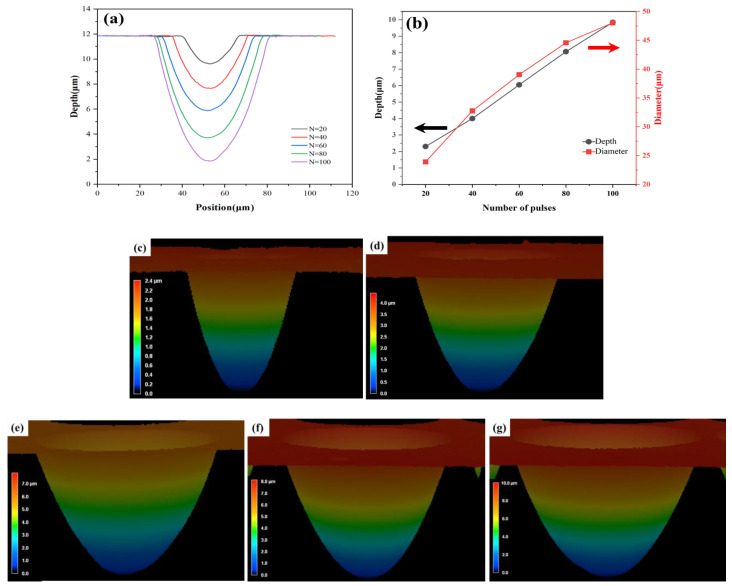
Controlled fabrication of micro lens profiles. (**a**) Profile curves of micro lens fabricated under different pulse numbers; (**b**) depth and diameter of the micro lens fabricated under different pulse numbers. The black arrow represents the depth curve of the left coordinate axis, and the red arrow represents the diameter curve of the right coordinate axis; (**c**–**g**) three-dimensional profiles of micro lens fabricated under different pulse numbers: (**c**) 20 pulses; (**d**) 40 pulses; (**e**) 60 pulses; (**f**) 80 pulses; (**g**) 100 pulses.

Furthermore, we prepared individual micro lenses. Under constant laser energy conditions, the formation of micro lens contours can be precisely regulated by varying the number of laser pulses. As depicted in Figure 4a,b, the depths of the micro lenses increase linearly with pulse numbers, and the measurement results are 2.3 μm, 4.05 μm, 6.05 μm, 8.06 μm, and 9.84 μm, corresponding to pulse numbers of 20, 40, 60, 80, and 100, respectively. Similarly, the diameter of the micro lens also increases approximately linearly with an increase in the number of pulses. Under different pulse numbers (20–100), the diameters of the micro lens achieved are measured to be 23.91 μm, 32.76 μm, 39.05 μm, 44.57 μm, and 48.04 μm, respectively.

In addition, higher single pulse energies lead to an increased depth and width of the micro lens profiles. This is due to the enlarging of the laser processing area on the surface of fused silica, resulting in a larger etched area. Figure 4c–g present the three-dimensional morphology of an individual micro lens etched with pulse numbers ranging from 20 to 100. It is worth noting that regardless of the number of pulses, each micro lens exhibits smooth contours and excellent surface quality. This is the result of complete etching. That is to say, we have found suitable laser parameters and the wet etching time. By modulating the number of pulses, we can effectively prepare the expected size and shape of the micro lens without affecting its surface smoothness.

### 3.4. Fabrication of MLAs

**Figure 5:**
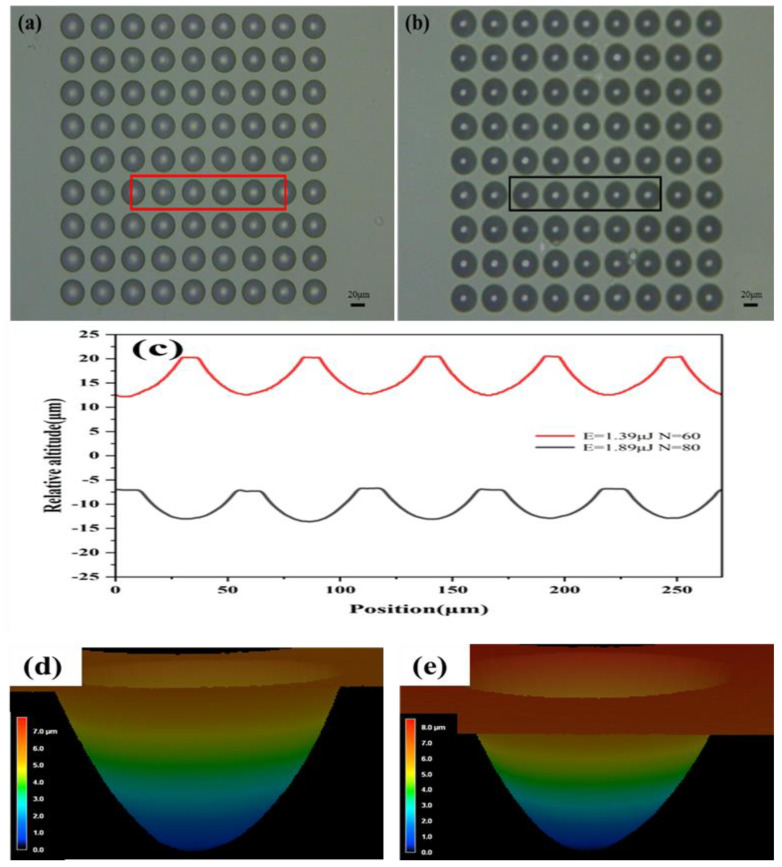
MLA schematics. (**a**) Micro lens array fabricated with a single pulse energy of 1.39 μJ and 60 pulses, scale bar: 20 μm; (**b**) micro lens array fabricated with a single pulse energy of 1.89 μJ and 80 pulses, scale bar: 20 μm; (**c**) profiles of the two micro lens arrays. The red curve represents the profile measurement result of the micro lenses within the red box in Figure 5a. The gray curve represents the profiles measurement result of the micro lenses within the gray box in Figure 5b; (**d**,**e**) three-dimensional views of individual micro lens structures from the two micro lens arrays.

Based on the aforementioned experimental findings, we successfully produced two types of 9 × 9 micro lens arrays, depicted in Figure 5a,b. Each MLA consists of 81 micro lenses, covering an area of approximately 0.16 mm^2^. The center-to-center spacing of each micro lens is 50 μm. The micro lens exhibits perfectly circular and uniform surface profiles. The contour measurements of these arrays are presented in Figure 5c. Among them, the red curve in Figure 5c shows the measurement result of the micro lens profiles in the solid box area of Figure 5a.

The results show that using a single pulse energy of 1.39 μJ and 60 pulses, the fabricated micro lenses have a diameter of approximately 39.7 μm and a depth of about 19.5 μm. Using the roughness measurement module of an LSCM, ten micro lenses are selected to measure the roughness of the bottom of the micro lenses (each measurement area is 1 μm × 1 μm). The measurement result shows that the average roughness is about 30 nm. The gray curve in Figure 5c, corresponding to the measurement result within the solid box area of Figure 5b, reveals that using a single pulse energy of 1.89 μJ and 80 pulses, the fabricated micro lens has a diameter of about 44.2 μm and a depth of approximately 22.8 μm, with an average surface roughness of approximately 50 nm (the measurement method is the same as above). Obviously, as the power and number of pulses increase, the depth and diameter of the implemented micro lens array also increase accordingly, as we have learned from our previous process research. Figure 5d,e depict the three-dimensional morphology of a representative micro lens from each array type captured under laser scanning confocal microscopy. Both arrays demonstrate exceptional surface morphology, smoothness, and uniformity. In both arrays, each lens exhibits a consistent geometric shape without any obvious visual defects. These superior characteristics are attributed to the minimal scattering observed during femtosecond laser processing and the meticulous optimization of process parameters [36].

### 3.5. Imaging Performance Test of MLAs

As we know, the image plane of a concave lens is in the negative direction. The expressions for the focal length *f* and numerical aperture (*NA*) that determine the imaging performance of micro concave lenses are as follows [6]:

(1) $$f=-\frac{h^{2}+{\left(\frac{D}{2}\right)}^{2}}{2h\left(n-1\right)}$$

(2) $$NA=\frac{D}{2\left|f\right|}$$

where *h* is the height of the micro lens, *D* is the diameter, *n* is the refractive index of the micro lens material, *f* is the focal length, and *NA* is the numerical aperture of the micro lens. The focal lengths of the micro lens arrays in Figure 5a,b are calculated to be −43.303 μm and −48.223 μm, respectively. It is worth noting that the numerical aperture (*NA*) of both MLAs is 0.458. That is to say, this study can also achieve a micro lens array with a constant aperture and variable focal length.

**Figure 6:**
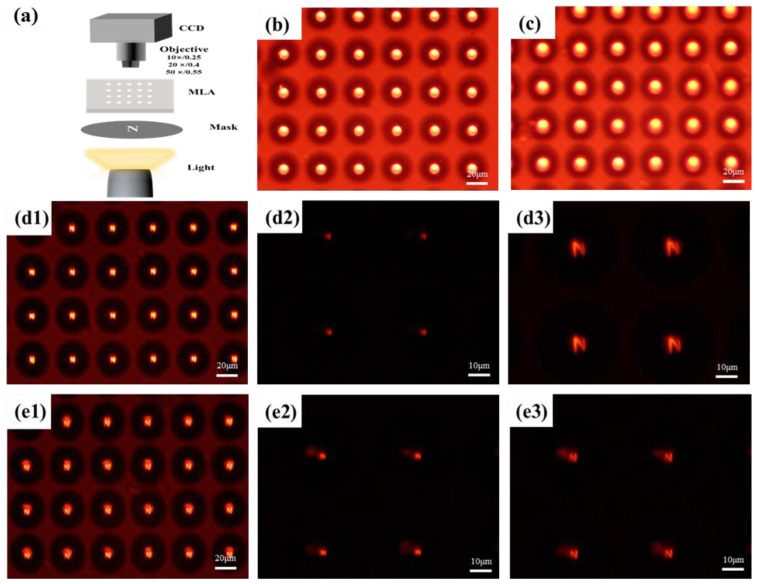
Imaging performance testing of the two MLAs shown in Figure 5a,b. (**a**) Optical path diagram of the imaging analysis system. The system is equipped with optional imaging objectives; (**b**,**c**) place the MLA shown in Figure 5a,b in the imaging analysis system, respectively. The CCD imaging results without the mask using an imaging objective of 20×. The scales are 20 μm; (**d1**–**d3**) CCD imaging results of the MLA shown in Figure 5a after inserting the mask N. The objective lenses used are 20×, 20×, and 50× in sequence. Among them, Figure 6(**d3**) is the imaging pattern obtained by moving the mask upwards based on Figure 6(**d2**). (**e1**–**e3**) CCD imaging results under the MLA shown in Figure 5b using the same testing method, corresponding to Figure 6(**d1**)–(**d3**). The scales in Figure 6(**d1**,**e1**) are 20 μm. The scales in Figure 6(**d2**,**d3**) and Figure 6(**e2**,**e3**) are 20 μm.

Finally, imaging performance tests were conducted with the optical setup depicted in Figure 6a. A halogen lamp served as the illumination source, directing light through a brown mask featuring a transparent letter ‘N’ (approximately 7 mm in size on the letter mask). The MLAs fabricated focused this light onto an objective lens positioned opposite, enabling a CCD to capture bright-field and dark-field images of the letter ‘N’ on the pseudo-focal plane of the MLAs without inserting the letter mask, and we observed the transparency of two manufactured MLAs, as shown in Figure 6b,c. Obviously, each micro lens structure in the array has good transparency and exhibits excellent roundness in their contours. When inserting the letter mask template, the imaging effects of the two MLAs are shown in Figure 6(d1–d3) and Figure 6(e1–e3), respectively. Both demonstrate clear imaging capabilities overall, with each micro lens exhibiting nearly consistent imaging performance within the same array, which indirectly indicates excellent uniformity of the array structure. Using a higher magnification 50× objective lens for local observations, it was found that the imaging performance of a single micro lens is also satisfactory, as depicted in Figure 6(d2,e2). By shifting the letter mask upward to adjust the relative distance between the mask and MLA, the imaging effect of the letter “N” can be further magnified, while maintaining clear imaging quality, as shown in Figure 6(d3,e3). Compared with Figure 6(d2), the imaging magnification of Figure 6(d3) has increased by about 3.0 times. In addition, the imaging magnification of Figure 6(e3) has increased by about 1.3 times compared to Figure 6(e2).

## 4. Conclusions

In summary, further improving the controllability of MLA preparation technology has become urgent in the new generation of MOSs. To achieve this, we introduced an innovative method called femtosecond laser penetration-induced modification-assisted wet etching (FLPIM-EL). Utilizing this technique, we successfully fabricated MLAs of tens of microns on the surface of fused silica. These micro lenses exhibited excellent morphology and smooth surfaces. In the first step, known as step 1-FLPIM, a penetrating induced modification method was adopted. This approach effectively mitigated the absorption and scattering effects caused by the modified layer from previous laser pulses, enhancing the overall controllability of the process. In step 2, an HF solution was utilized to wet etch the modified layer completely. By adjusting the single pulse energy and pulse number of the laser, the size of the laser-modified regions was precisely controlled, thereby regulating the dimensions and depths of the micro lens. This provides a viable approach to creating micro lens arrays with a constant NA with variations in the focal length, specifically, as follows:

In this paper, we have achieved two different types of 9 × 9 MLAs on fused silica with the FLIM-EL technique: one using 60 pulses with an energy of 1.39 μJ, and the other using 80 pulses with an energy of 1.83 μJ. The depths of the two MLAs were 19.5 μm and 22.8 μm, respectively, and the heights were 39.7 μm and 44.2 μm, respectively. The surface roughness was as low 30 nm. It is important to note that the two MLAs have the characteristic of a fixed NA (0.458) but different focal lengths (−43.303 μm and −48.223 μm). Meanwhile, the MLAs exhibits significant uniformity and excellent imaging performance, enabling the clear imaging of mask patterns at adjustable magnifications (1.3×–3.0×). This study will further advance the potential applications of tunable MOSs. For example, in AR/VR devices, it is used to enhance the depth of the field effect and achieve a more natural virtual scene interaction; in optical fiber communication, it is used to optimize the coupling efficiency of optical signals, thereby improving the reliability of signal transmission.

## Data Availability

The original contributions presented in the study are included in the article; further inquiries can be directed to the corresponding author/s.
